# Effect of Covalence and Degree of Cation Order on the Luminous Efficacy of Mn^4+^ Luminescence in the Double Perovskites, Ba_2_*B*TaO_6_ (*B* = Y, Lu, Sc)

**DOI:** 10.1021/acs.jpclett.4c00205

**Published:** 2024-04-10

**Authors:** Alok M. Srivastava, Mikhail G. Brik, Chong-Geng Ma, William W. Beers, William E. Cohen, Michal Piasecki

**Affiliations:** † Current Chemicals, 1099 Ivanhoe Road, Cleveland, Ohio 44110, United States; ‡ School of Optoelectronic Engineering & CQUPT-BUL Innovation Institute, Chongqing University of Posts and Telecommunications, Chongqing 400065, PR China; § Center of Excellence for Photoconversion, Vinča Institute of Nuclear Sciences, National Institute of the Republic of Serbia, University of Belgrade, Belgrade 11351, Serbia; ∥ Institute of Physics, University of Tartu, W. Ostwald Str. 1, Tartu 50411, Estonia; ⊥ Academy of Romanian Scientists, Ilfov Str. No. 3, Bucharest 050663, Romania; # Faculty of Science and Technology, Jan Długosz University, Armii Krajowej 13/15, Czestochowa 42200, Poland

## Abstract

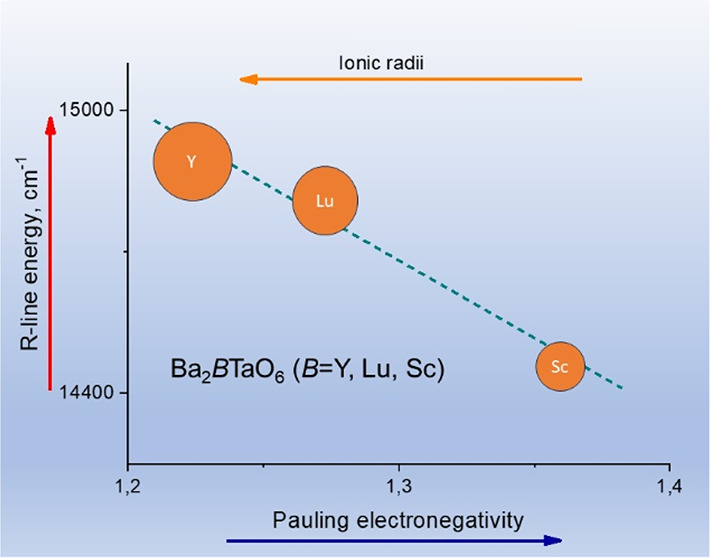

The spectroscopic properties of the Mn^4+^ ion are investigated in the series of isostructural double perovskite compounds, Ba_2_*B*TaO_6_ (*B* = Y, Lu, Sc). A comparison of these properties highlights the influence of covalent bonding within the perovskite framework and the degree of order between the B^3+^–Ta cations on the energy and intensity of the Mn^4+2^E → ^4^A_2_ emission transition (R-line). These two parameters of the emission spectrum are of importance for practical application since they determine the phosphor luminous efficacy. The influence of covalent bonding within the corner shared BO_6/2_ and TaO_6/2_ perovskite framework on the energy of the R-line energy is investigated. From the spectroscopic data, we have derived information on the influence of the degree of order between the B^3+^ and Ta^5+^ cations on the intensity of the R-line. The lowest energy and the highest intensity of the R-line are found in the double perovskite, Ba_2_ScTaO_6_. The purpose of this work is to propose for first time an explanation of these effects in the considered double perovskites. The obtained results are useful guidelines for practical improvement and tuning of key parameters of phosphors to the desired values.

The modern-day phosphor converted light emitting diodes (pc-LEDs) are based on the blue emitting InGaN chip coated with a phosphor or a blend of phosphors that produces light of a specific spectral power distribution. The energy saving and cost-effective pc-LED technology has been developed for use in such diverse applications as displays, general illumination, traffic signals and horticultural lighting to name a few. The commercial success of the red emitting K_2_SiF_6_:Mn^4+^ (KSF/PFS) phosphor for general illumination application is traced to the sharp line emission spectrum that matches well with the human eye sensitivity curve. This provides for the construction of white light emitting diodes with high efficacy and good color rendering index.^1^ The enormous market potential for such pc-LED devices has prompted the fundamental studies of the Mn^4+^ ion luminescence in diverse compounds such as fluoride, oxides, and oxyfluoride.^1−5^ These ongoing fundamental inquiries are taking place at a very rapid pace.

The luminescence of the Mn^4+^ ion, with the 3d^3^ electronic configuration, is composed of the ^2^E → ^4^A_2_ sharp line transition (R-line; zero-phonon line or ZPL) accompanied by the Stokes and anti-Stoles vibronic sidebands. The emission spectrum of the Mn^4+^ ion can be tuned to satisfy the requirement of a particular pc-LED device. For general illumination application, the phosphor must generate high luminous efficacy (lumens per watt or LPW). High LPW is attained when the emission spectrum makes a good match with the human eye response function (luminosity response function). Our fundamental studies have established two factors of importance for high LPW, (1) the energy (emission wavelength) and (2) the intensity of the Mn^4+^ spin-forbidden ^2^E → ^4^A_2_ emission transition.^1,6^ The phosphor luminous efficacy is maximized in host crystals which support high energy and high intensity of the R-line. Therefore, it is instructive to inquire about the properties of the host lattice that maximize the luminous efficacy.

In the Tanabe-Sugano diagram for the ions with d^3^ electron configuration, the energy of the ^2^E state is independent of the crystal field strength (*Dq*). The energy of the ^2^E → ^4^A_2_ emission transition is determined chiefly by the covalence of the “Mn^4+^–ligand” bonding.^7−9^ A blue or a red shift of the emission spectrum can be induced by altering the bonding covalence. In highly ionic compounds such as fluorides, the emission is blue-shifted relative to oxidic compounds with higher covalent character.

The intensity of the R-line is determined by the site symmetry: the higher the point symmetry of the impurity ion site, the lower is the R-line intensity, and vice versa.^6^ The presence or absence of an inversion center at the Mn^4+^ ion site is key to the strength of the R-line. In a site with inversion symmetry, the parity selection rule cannot be lifted by the odd-parity crystal field components. In such sites, the R-line has magnetic dipole character with low oscillator strength. In this case, the R-line has weak intensity relative to the vibronic sidebands which have a much larger oscillator strength since they are electric-dipole allowed transitions. In sites lacking inversion symmetry, the R-line gains intensity relative to the vibronic sidebands due to the relaxation of the parity selection rule. Increase in R-line intensity has important practical consequences since it causes color changes which increase the phosphor luminous efficacy.^1,6^

In a site where the Mn^4+^ ion has near or ideal octahedral symmetry with respect to the nearest neighbor anions, it is possible to increase the R-line intensity by deliberately creating cationic disorder in the second coordination sphere (next-nearest neighbor cations). We explored this approach to increasing R-line intensity with solid solution systems that provided random cation distribution in compounds crystallizing with the pyrochlore^10^ and double perovskite structures.^11^ The site disorder in the second coordination sphere increased the R-line intensity by lowering the symmetry around the Mn^4+^ ion. Different cations in the second coordination sphere with different ionic radii and electronegativities can alter Mn^4+^–ligand bond distances and ligands’ effective charges. These changes in the geometric and electronic parameters can remove the inversion center in the impurity cluster. More recently, we reported on the influence of deliberately induced cationic disorder on the optical properties of Cr^3+^ in spinel oxides.^12^

It is of fundamental and practical interest to inquire about the structural and chemical bonding factors which influence the energy and intensity of the Mn^4+2^E → ^4^A_2_ emission transition. Our approach to this inquiry is to investigate the influence of compositional variations on the spectroscopic properties of the Mn^4+^ ion in a series of isostructural compounds. In the spirit of this approach, we have reported on the luminescence of Mn^4+^ ion in double perovskite compounds with varying compositions.^5,11,13−15^ Such studies unravel composition–optical properties relationships. In this work, we report on a comparative study of the spectroscopic properties of the Mn^4+^ ion in a series of double perovskite compounds with the general formula Ba_2_*B*TaO_6_ (*B* = Y, Lu, Sc). The study highlights the influence of bonding covalence and degree of *B*–Ta cation mixing on the luminescence of the Mn^4+^ ion.

The synthesized material in powder form was examined by X-ray diffraction to determine the crystal structure and lattice site occupation. The double perovskite A_2_*BB*′O_6_ is derived from the simple perovskite, A*B*O_3_, by ordering of *B* and *B*′ cations in alternate octahedral sites. The Ba_2_*B*TaO_6_ (*B* = Y, Lu, Sc) compounds crystallize in the cubic structure with space group *Fm*3*m*. In Figure 1 are shown the unit cell of Ba_2_YTaO_6_ and the characteristic linear [−Y–O–Ta−] linkage. In the fully ordered arrangement, each Y cation has only Ta as the next nearest neighbor and vice versa. However, this ordering is never complete, and some degree of mixing between the *B* and Ta cations occurs. The degree of order is determined by the difference between the ionic size and charge of the *B* and Ta cations.^16^ It is also determined by the process employed in the synthesis of these compounds.

**Figure 1:**
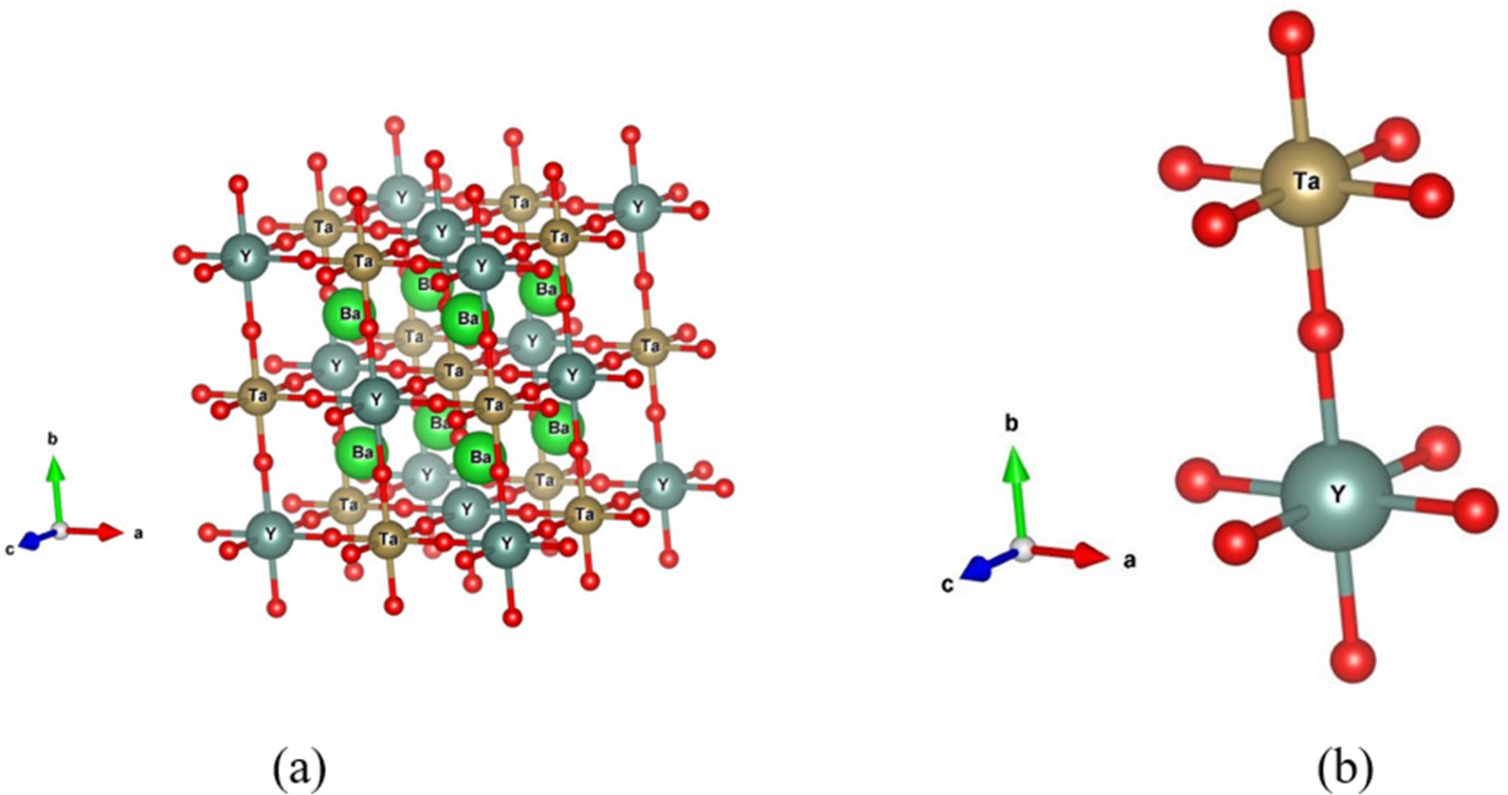
(a) The unit cell of Ba_2_YTaO_6_ and (b) the linear [Y–O–Ta] linkage. Drawn by VESTA.^17^

The Goldschmidt geometric tolerance factor *t* for the double perovskite with the general formula *A*_2_*BB*′*O*_6_ can be written as^16^
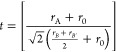
1where *r*_A_ is the ionic radii of the A cation in 12-fold coordination, *r*_*B*_ and *r*_*B*′_ are the ionic radii of *B* and *B*′ ions in 6-fold coordination, and *r*_0_ is the ionic radii of the oxygen ion (1.40 Å). The value of *t* reflects the stability of the perovskite structure. The highest stability occurs for *t* = 1, which corresponds with the ideal perovskite structure. The tolerance factor for Ba_2_*B*TaO_6_ compounds, listed in Table 1, is close to 1. This is consistent with their cubic symmetry as determined by X-ray diffraction. It implies that the *B*–O–Ta bonds are 180° (linear) and the *B*O_6/2_ and TaO_6/2_ units are perfect octahedrons.

**Table 1:** Crystallographic Data of Cubic (Space Group *Fm*3*m*) Double Perovskites Ba*B*TaO_6_ (*B* = Y, Lu, Sc)^a^

	Lattice constant (Å)	Unit cell volume (Å^3^)	*B*-0 (Å)	*Ta*-0 (Å)	*R*_*B*^3+^_ (Å)	*t*
Ba_2_YTaO_6_	8.43539	600.23	2.232	1.986	0.90	0.98
Ba_2_LuTaO_6_	8.3760	587.64	2.203	1.985	0.861	0.99
Ba_2_ScTaO_6_	8.226	556.5	2.0894	2.0236	0.745	1.02

a *B*-*0* and *Ta*-0 are the bond distances (Å). *R*_*B*^3+^_ is Shannon’s ionic radii of six coordinated *B* cations.^20^ The data for Ba_2_ScTaO_6_ are from ref (20). The data for *B* = Y, Lu are from ICSD. *t* is the tolerance factor calculated using eq 1.

The double perovskite compounds were found to be single phase materials by their X-ray diffraction patterns. As an example, we exhibit the X-ray diffraction pattern of the Ba_2_Y(Ta_0.99_Mn_0.01_)O_6_ compound in Figure 2.

**Figure 2:**
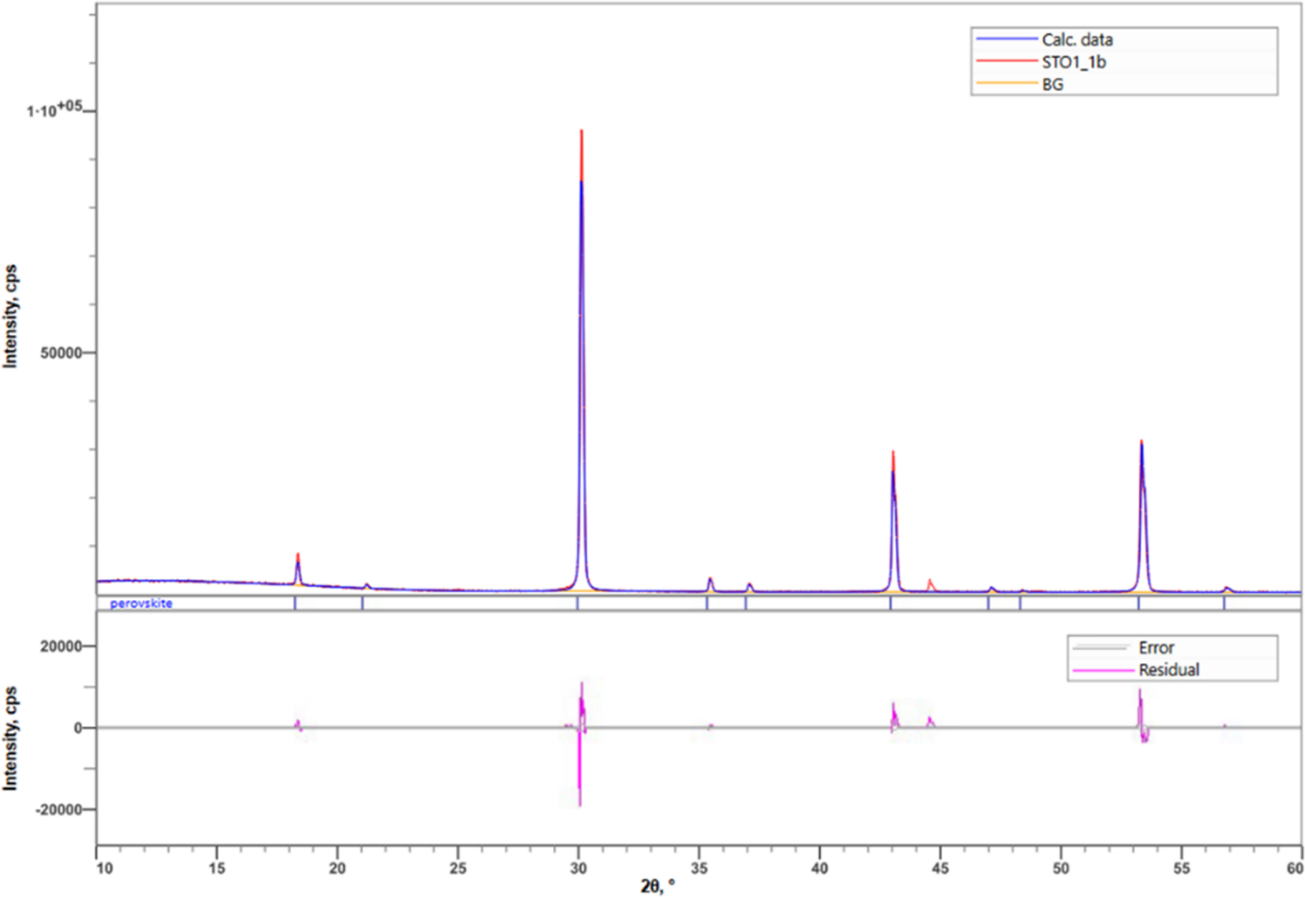
X-ray diffraction powder pattern of cubic Ba_2_Y(Ta_0.99_Mn_0.01_)O_6_. The sample was found to be phase-pure.

The crystallographic data for the double perovskites that are relevant to our discussion are assembled in Table 1. We have indicated in the Introduction that a goal of this work is to inquire about the influence of the degree of order between *B*–Ta cations on the Mn^4+^ luminescence. This ordering is never complete and is driven by the differences between the charges and ionic radii of the cations.^16^ Cationic disorder can affect the unit cell volume of the double perovskite compound. For example, if Ta^5+^ replaces a *B*-cation in the [*B*–O–Ta] linkage of Ba_2_*B*TaO_6_, then it will have another highly charged Ta^5+^ as its next neighbor cation (see Figure 1). The resulting electrostatic repulsion due to this antisite disorder will increase the lattice constant or the unit cell volume. To check for any evidence of *B*–Ta antisite disorder within the available crystallographic data, the unit cell volume of Ba_2_*B*TaO_6_ (*B* = Y, Lu, Sc) as a function of the *B*-cation ionic radii was plotted (Figure 3). The ionic radii are tabulated by Shannon.^18^ We can see that the cell volume increases linearly as ionic radii of the *B*-cation increase. Therefore, any *B*–Ta antisite disorder is not seen in the lattice constant data that are obtained by X-ray diffraction. As we will show in the later sections, the luminescence data are more sensitive techniques for probing order–disorder phenomena.

**Figure 3:**
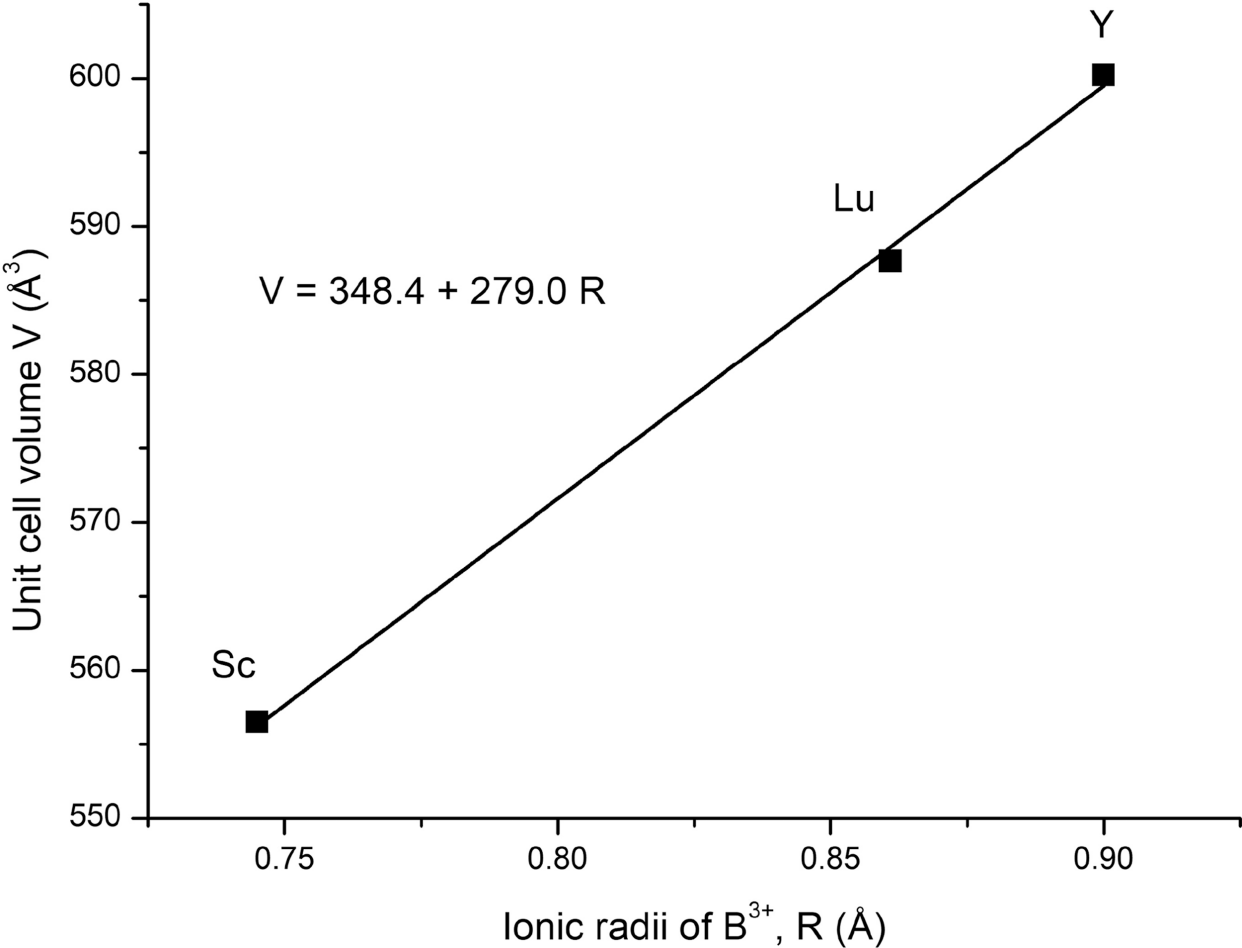
Unit cell volume plotted against the ionic radii of *B*^3+^ cation for Ba_2_*B*TaO_6_ (*B* = Y, Lu, Sc).

The interpretation of the optical data requires the knowledge of the host lattice site substituted by Mn^4+^. In these double perovskite compounds, there are two octahedral sites available for substitution. Note from the Experimental Section that the compositions are formulated as Ba_2_*B*(Ta_0.99_Mn_0.01_)O_6_. Nonetheless, it may be argued that in the synthesized sample, the substitution 2Mn^4+^ → *B*^*3+*^ + Ta^5+^ is favored since it ensures charge balance.

However, ionic radii consideration reasons against such substitution. The ion size difference between Mn^4+^ (0.54 Å) and Sc^3+^ (0.745 Å), which is the smallest *B* cation in this series of compounds, is ∼40%. In contrast, the ion size mismatch between Mn^4+^ and Ta^5+^ is ∼20%. To minimize local elastic strain effects or lattice distortions, the impurity Mn^4+^ ion will preferentially substitute at the octahedral Ta^5+^ site in Ba_2_*B*TaO_6_ (*B* = Y, Lu, Sc). This reasoning is supported by computational modeling of the site occupation and defect formation in the isostructural niobate, Ba_2_YNbO_6_:Mn^4+^. The result shows that the occupation of the Nb^5+^ site is preferred with the charge compensation taking place via an antisite disorder (using the Kröger-Vink notation):^19^
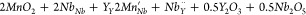


The low temperature (*T* = 12 K) excitation spectra of Ba_2_*B*TaO_6_:Mn^4+^ (*B* = Y, Lu, Sc,) are shown in Figure 4. The excitation spectra consist of four major bands corresponding with the host lattice absorption (HL), the O^2–^ → Mn^4+^ charge transfer transition (CT), and the spin-allowed but parity-forbidden (as taking place between the states of the 3d^3^ electron configuration without changing the states’ parity) ^4^A_2_ → ^4^T_1_ and ^4^A_2_ → ^4^T_2_ transitions. In Table 2 are assembled the transition energies along with the room temperature data that are available on two of these double perovskite compounds in archival literature.

**Table 2:** Energies of the Mn^4+^ Transitions in the Excitation Spectra of Ba*B*TaO_6_ (*B* = Y, Lu, Sc) at *T* = 12 K^a^

	HL	CT	^4^A_2_ → ^4^T_1_	^4^A_2_ → ^4^T_2_	*Dq*	Ref
Ba_2_YTaO_6_	34 842	30 581	28 570	19 230	1923 (1879)	This work
Ba_2_YTaO_6_	35 000	30 810	27 745	18 950	1895	(21)
Ba_2_LuTaO_6_	34 129	30 248	28 012	18 996	1899 (1832)	This work
Ba_2_LuTaO_6_	34 129	30 303	27 397	18 518	1852	(22)
Ba_2_ScTaO_6_	33 333	29 154	25 303	17 857	1785 (1715)	This work

a HL is host lattice absorption; CT is the O^2–^ → Mn^4+^ charge transfer transition. The room temperature *Dq* values are in parentheses. The literature data for Ba_2_YTaO_6_^21^ and Ba_2_LuTaO_6_^22^ are at room temperature. All values in cm^–1^.

**Figure 4:**
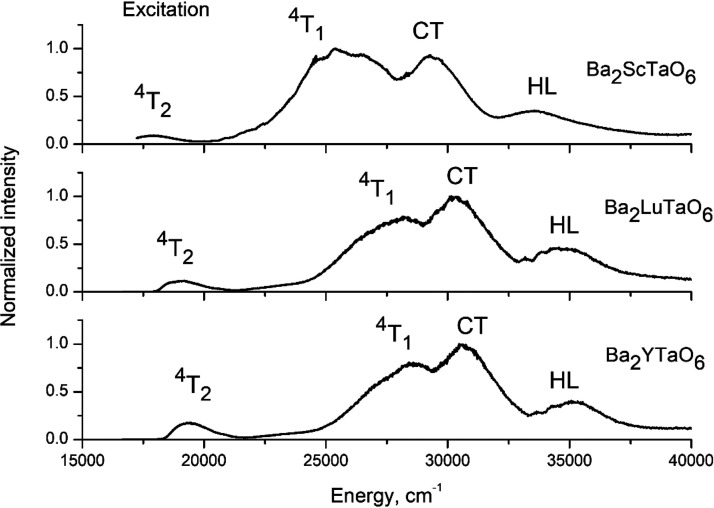
Excitation spectra for Mn^4+^ luminescence of Ba_2_YTaO_6_ (λ_em_= 685 nm), Ba_2_LuTaO_6_ (λ_em_= 689 nm), and Ba_2_ScTaO_6_ (λ_em_= 708 nm) at *T* = 12 K.

We first discuss the compositional dependence of the host lattice absorption energy (HL). The absorption is due to the O^2–^ → Ta^5+^ charge transfer transition which involves the transfer of an O-2p electron to the empty t_2g_ (π-bonding) orbital of the Ta^5+^ ion. It increases in the order, Sc < Lu < Y and is, therefore, correlated with the *B*-cation. Band gap energies of Ba_2_ScTaO_6_ and Ba_2_YTaO_6_, which are obtained from band-structure calculations, are 3.35 eV (27 019 cm^–1^) and 3.99 eV (32 181 cm^–1^), respectively.^23^ The observed trend can be rationalized from the results of electronic structure calculations of the compounds, which establish the degree of covalency (hybridization) between the orbitals of *B*–Ta cations and oxygen 2p orbitals. It should be noted that due to the ordering of *B* and Ta cations, each oxygen ion will have Ta^5+^ with the 5d^0^ electronic configuration on one side and the *B*-cation on the other side (Figure 1). In an octahedral coordination, the crystal field splits the 5-fold degenerate (in the free ion) d levels into lower 3-fold-degenerate t_2g_ (*d*_*xy*_,*d*_*yz*_,*d*_*xz*_) and the upper 2-fold-degenerate e_g_ (*d*_*z*^2^_,*d*_*x*^2^-*y*^2^_) levels. The Ta–O bonding involves the hybridization between the O 2p_π_ and the Ta t_2g_ orbitals.

The band-structure of Ba_2_ScTaO_6_ has been reported.^20^ The Sc^3+^ ion, with the 3d^0^ electronic configuration, has empty d orbitals for covalent bonding with the O 2p_π_ orbitals. Therefore, in the [ScTaO_6_] framework of Ba_2_ScTaO_6_, the π bonding between O 2p_π_ orbitals and Sc/Ta t_2g_ orbitals occurs on both side of each oxygen ion. This bonding character is reflected in the calculated band-structure. The conduction band is a mixture of Sc 3d and strongly hybridized Ta–O antibonding states. The conduction band edge is composed of the Ta 5d orbitals because the Ta–O hybridization is stronger than the Sc–O hybridization. This is because of the following factors: (1) the higher electronegativity of Ta (1.5 on the Pauling scale) relative to Sc (1.36), (2) the larger radial extension of the 5d orbital than the 3d orbital, and (3) the smaller Ta–O bond distance. The consequence of stronger Ta–O hybridization is the larger crystal field splitting (the t_2g_–e_g_ separation) of the Ta 5d state (∼5.5 eV) than that of the Sc 3d state (∼2.2 eV). For this reason, the edge of the conduction band is composed of the Ta 5d orbitals.

The band-structure of Ba_2_YTaO_6_ shows a relatively smaller energetic overlap between the Y 4d and the O 2p states.^23^ The calculated band-structure of the Lu-phase is like that of the Y-phase.^24^ In these compounds, the π bonding with the Ta t_2g_ orbital occurs only on one side of each oxygen ion. The structural and electronic isolation of the TaO_6_ octahedron appreciably narrows the conduction band and increases the band gap of Ba_2_YTaO_6_ relative to Ba_2_ScTaO_6_. We digress briefly to point out that the narrowing of the conduction band in compounds with the double perovskite structure has a profound influence on the optical properties of impurity ions. The conduction band narrowing that resulted from the isolation of the TiO_6_ octahedral group and the deviation of the Mg–O–Ti bond angle from the ideal 180° in the double perovskite La_2_MgTiO_6_ was responsible for first observation of the localized Bi^3+3^P_0,1_→ ^1^S_0_ emission in a titanate host.^25,26^

As suggested in ref (23), the band gap of these double perovskite compounds is an accurate indicator of the O^2–^ → Ta^5+^ charge transfer energy (HL). Figure 5 shows that HL decreases almost linearly as the electronegativity of the *B*-cation increases (on the Pauling scale). This leads to the conclusion that the covalent bonding within the perovskite framework of Ba_2_*B*TaO_6_ increases in the sequence, Y< Lu < Sc. That the scandate perovskite is most covalent is not surprising since the Sc^3+^ ion, with the 3d^0^ electronic configuration, has empty d-orbitals for covalent bonding with the oxygen anion, as discussed previously.

**Figure 5:**
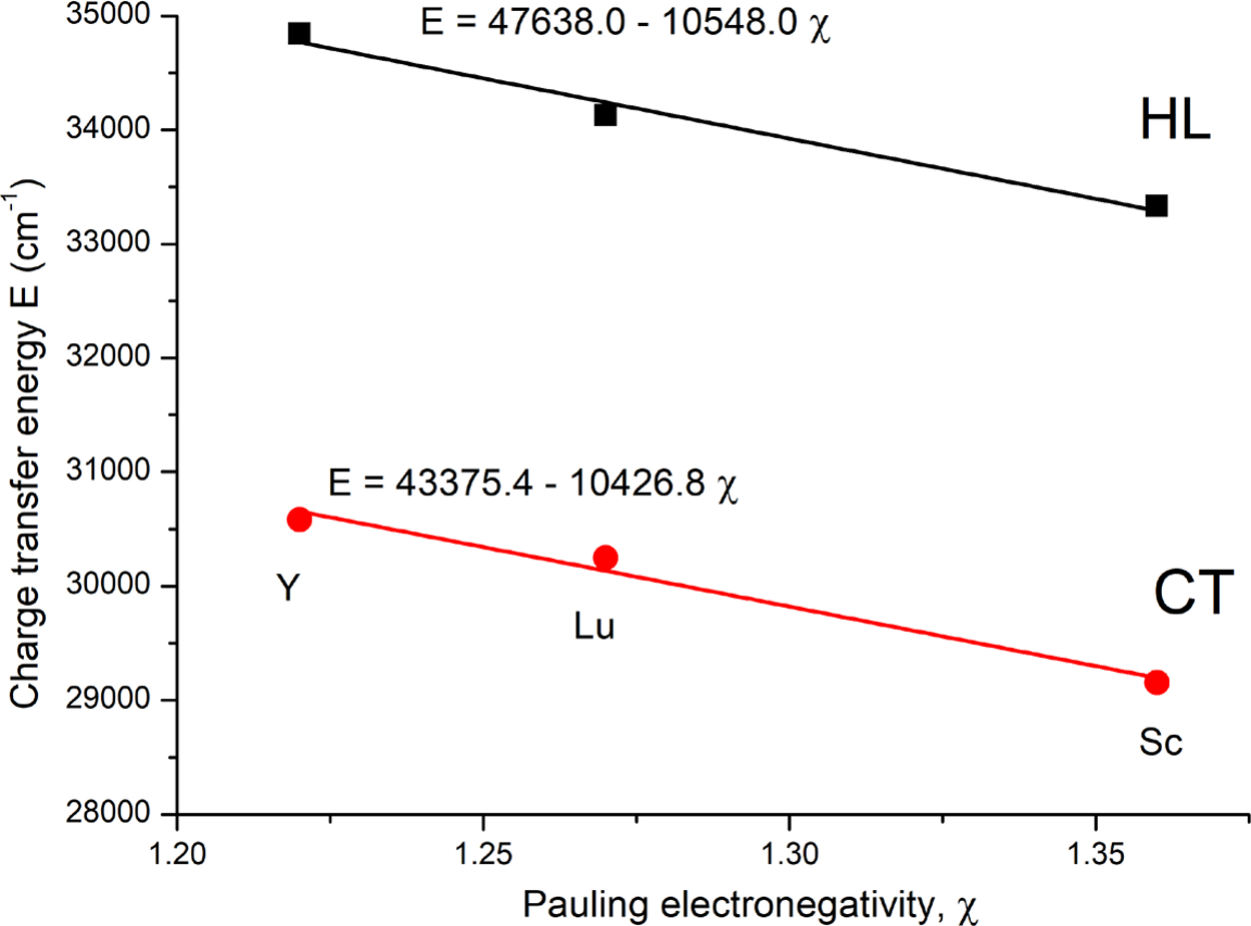
Relationship between the energies of the host lattice absorption (HL; O^2–^ → Ta^5+^ charge transfer) and the O^2–^ → Mn^4+^ charge transfer transition (CT) and the electronegativity of the *B*-cation (on the Pauling scale) in Ba_2_*B*TaO_6_ (*B* = Y, Lu, Sc).

The compositional dependence of the CT is also associated with the *B*-cation electronegativity (Figure 5). Furthermore, both the HL and the CT decrease at the same linear rate with respect to electronegativity and framework covalency. Thus, the energy difference between the O 2p and the Mn^4+^ 3d orbitals decreases as the covalence of perovskite framework increases in the Ba_2_*B*TaO_6_ compounds.

The energy difference between the ^4^A_2_ ground and ^4^T_2_ excited states is the strength of the octahedral crystal field, Δ_o_ = 10 *Dq*. The values of *Dq* are calculated by the formula, E(^4^A_2_ →^4^T_2_)/10 = *Dq*, where E(^4^A_2_ →^4^T_2_) is the peak energy of the optical transition. The point charge model of crystal field predicts *Dq* to vary as R^–5^, where R is the “Mn^4+^–ligand” bond distance. In real systems, the power of R can be considerably different due to factors such as covalence which is neglected in the point charge model.^27^ The experimental *Dq* parameters can be analyzed in terms of the Ta–O bond distances. Since the local relaxation around the impurity ion will be different in the three perovskite compounds, the relaxed Mn–O bond distances R(Mn–O)) were calculated from the Ta–O bond distances (R(Ta–O)) and the ionic radii of Mn^4+^ (*r*_*Mn*^4+^_), Ta^5+^ (*r*_*Ta*^5+^_), and oxide (*r*_*o*^2–^_) ions by the formula:
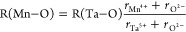
2

The relaxed R(Mn–O) bond distances are 1.878 Å for the Y/Lu-phases and 1.976 Å for the Sc-phase. The lower *Dq* = 1785 cm^–1^ for the scandate perovskite is consistent with the longer R(Mn–O) relative to the Y (*Dq* = 1923 cm^–1^; 1.878 Å) and Lu (*Dq* = 1899 cm^–1^; 1.878 Å)-phases. Note that despite the same R(Mn–O), *Dq* is slightly smaller in Ba_2_LuTaO_6_ than that in Ba_2_YTaO_6_. This shows the effect of the next nearest neighbor cations on *Dq*. In the [−*B*–O–Mn−] linkage, the Lu^3+^ with the smaller ionic radii will bond more strongly with the oxide ion than the larger Y^3+^ ion. The formal charge on the O^2–^ is expected to be smaller for *B* = Lu, which decreases *Dq*. Also given in Table 2 are values of *Dq* at room temperature. The increase in the strength of the crystal field at low temperature is due to shortening of the Mn–O bond by thermal contraction.

The pattern in the excitation spectra is different between the scandate and Y/Lu-phases. The O^2–^ → Mn^4+^ charge transfer transition (CT) is the most intense band in the excitation spectra of Y and Lu-perovskites (see Figure 4). In Ba_2_ScTaO_6_, the ^4^A_2_ →^4^T_1_ transition is relatively stronger. The lowest energy of the O^2–^ → Mn^4+^ transition is found in this perovskite (Table 2). An increase in nonradiative energy loss with decreasing ligand-to-metal charge transfer energy is not uncommon.^28^ Therefore, the radiative efficiency under O^2–^ → Mn^4+^ charge transfer excitation is lower since it provides for a more efficient nonradiative pathway for the excitation energy to decay directly to the ground state in the scandate perovskite.

The low temperature (*T* = 12 K) emission spectra of Ba_2_*B*TaO_6_:Mn^4+^ (*B* = Y, Lu, Sc,) are exhibited in Figure 6. At this temperature, the emission spectra are composed of the R-line and their associated Stokes vibronic bands, which correspond with the ungerade modes of the [MnO_6_] octahedral moiety.^3^ The energies of these lines are summarized in Table 3. The energy of the vibronic sidebands matches well with the data reported for [MnO_6_] moiety in the double perovskite, Ba_2_GdNbO_6_.^29^

**Table 3:** Energy of the R-Line and the Stokes Vibronic Lines in the Emission Spectra of Ba_2_*B*TaO_6_:Mn^4+^ (*B* = Y, Sc, Lu) at *T* = 12 K^a^

	R-line (ZPL)	Lattice modes	ν_6_ (t_2u_; bend)	ν_4_ (t_1u_; bend)	ν_3_ (t_1u_; stretch)
Ba_2_YTaO_6_	14 885	88/179/227	291	367	447/485
Ba_2_LuTaO_6_	14 788	104/177	287	366	450/499
Ba_2_ScTaO_6_	14 415	95/137/193	297	342/384	470
Ba_2_GdNbO_6_	15 059	-	280	364	434/449

a All values in cm^–1^. The data for Ba_2_GdNbO_6_ are from ref (29).

**Figure 6:**
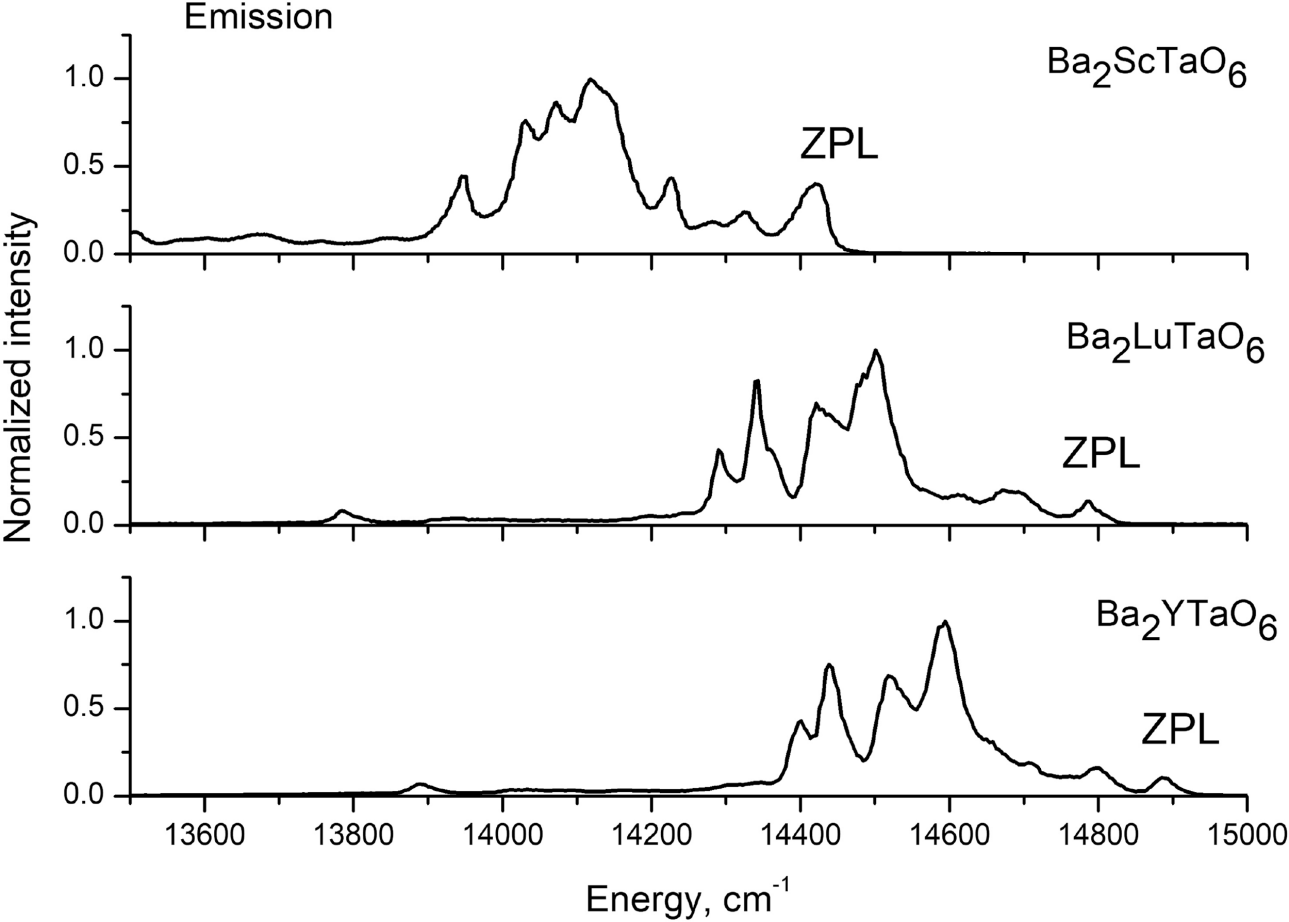
Emission spectra for Mn^4+^ luminescence of Ba_2_YTaO_6_ (λ_ex_= 330 nm), Ba_2_LuTaO_6_ (λ_ex_= 330 nm), and Ba_2_ScTaO_6_ (λ_ex_= 390 nm) at *T* = 12 K. The R-line (ZPL) is identified in the spectra.

The influence of covalency on the emitting ^2^E energy level is clearly seen in the emission spectra. In the Tanabe-Sugano diagram for the d^3^ configuration, the energy of ^2^E → ^4^A_2_ emission transition depends on the covalence of the Mn–O bonding.^7−9^ The data assembled in Table 3 indicate the following order of Mn–O bond covalency in Ba_2_*B*TaO_6_, Y< Lu < Sc. Generally, a specific parameter that influences the R-line energy in perovskite oxides is the deviation of the O–Mn–O bond angle from ideal octahedral 90°.^30,31^ The angular deviation decreases the hybridization between Mn^4+^ t_2g_ (dπ) and O 2p_π_ orbitals. The reduced Mn–O bond covalence raises the R-line energy. In cubic Ba_2_*B*TaO_6_ (*B* = Y, Lu, Sc), the Ta–O–Ta bond angles are perfect 90°.Therefore, the R-line energy is determined by the covalence of the perovskite framework, which increases in the same sequence, Y< Lu < Sc. As shown in Figure 7, the energy of the R-line decreases linearly with increasing electronegativity of the *B*-cation in Ba_2_*B*TaO_6_ (*B* = Y, Lu, Sc). The Mn^4+^ ion is most covalently bonded in the Sc-phase and least in the Y-phase.

**Figure 7:**
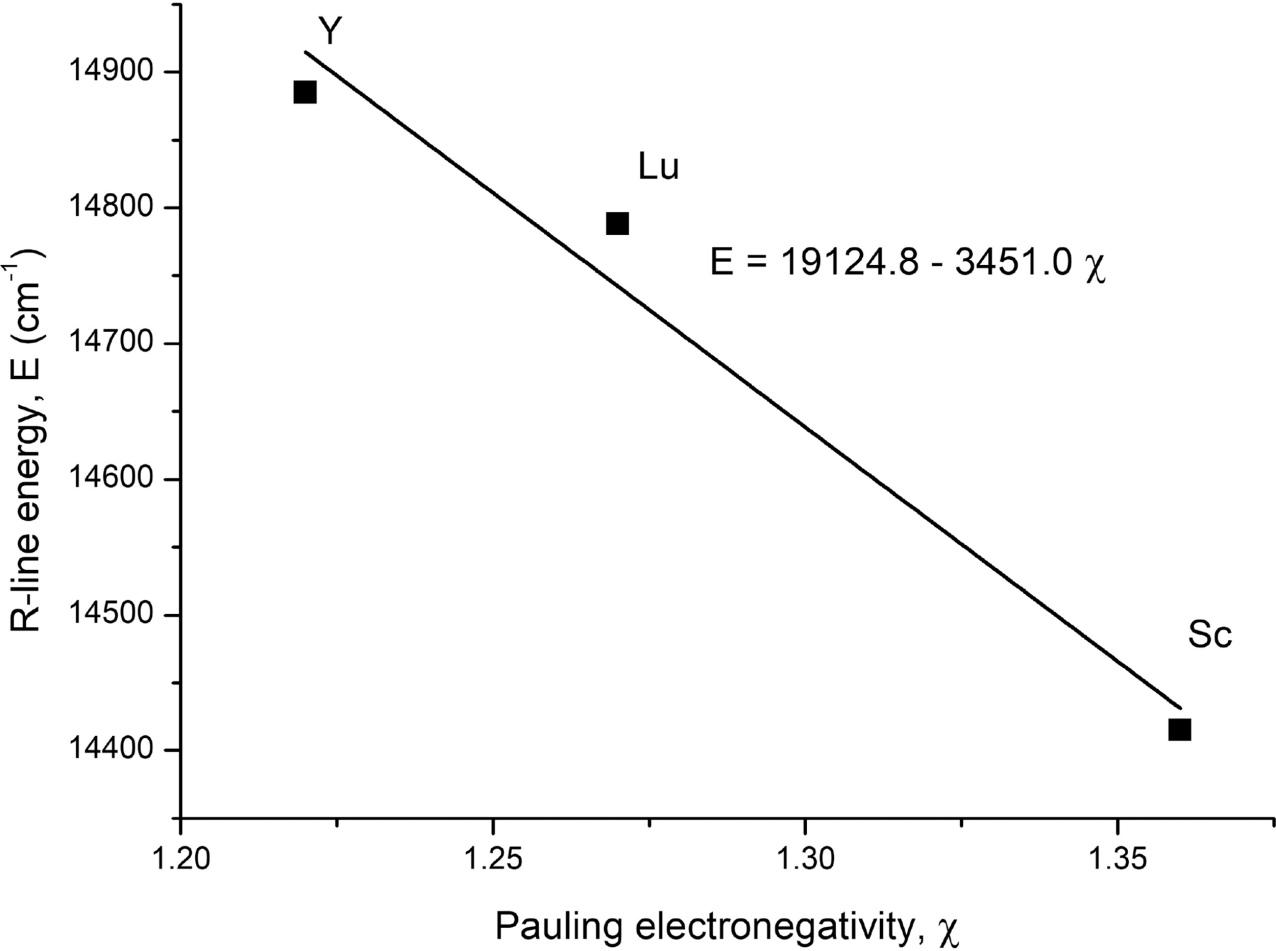
Relationship between the R-line energy and the electronegativity (on the Pauling scale) of the *B*-cation in Ba_2_*B*TaO_6_ (*B* = Y, Lu, Sc).

It is pointed out that while the R-line and the vibronic sidebands have sharp features in Ba_2_*B*TaO_6_ (*B* = Y, Lu), they are broadened in the Sc-phase. It is also pointed out that the R-line is considerably more intense in the Sc-phase than in the other two phases. These properties are accounted for in the next section.

In Table 4 are assembled the values of the Racah parameters *B*, *C* (along with the crystal field strength *Dq*) for the Mn^4+^ ion. The parameters were estimated from the excitation and emission spectra of the considered double perovskites with the help of standard equations for the energy levels of the d^3^ electron configuration.^32^ In addition, the values of the new nephelauxetic parameter (9) were calculated; *B*_0_ = 1160 cm^–1^, *C*_0_= 4303 cm^–1^ are the Racah parameters for the free Mn^4+^ ion.^33^

**Table 4:** Spectroscopic Parameters for the Mn^4+^ Ions (All in cm^–1^) in Ba*B*TaO_6_ (*B* = Y, Lu, Sc) Double Perovskites

	*B*	*C*	*Dq*	β_1_	Ref
Ba_2_YTaO_6_	1019	2560	1923	1.061	This work
Ba_2_LuTaO_6_	971	2627	1899	1.036	This work
Ba_2_ScTaO_6_	756	2958	1785	0.947	This work

The β_1_ parameter reflects the reduction of the Racah parameters caused by covalent bonding when the ion is embedded in a host compound.^9^ The smaller the value of β_1_, the higher is the covalence of the Mn–O bonding. The data indicate that the Mn–O bonding covalency increases in the sequence, Y < Lu < Sc. This is exactly as derived from the structural peculiarities of these double perovskite frameworks and influence of the second cation on the Mn^4+^ spectroscopic properties.

In the next step, we show that the intensity of the Mn^4+^ R-line is correlated with the degree of order between the *B*–Ta cations in Ba_2_*B*TaO_6_. In Table 5 are listed the peak intensity ratios of the R-line to its most intense Stokes ν_6_ sideband (R_I_). Also listed are the ionic radii differences between the *B*–Ta cations. The Shannon ionic radius of 6-fold coordinated Ta^5+^ ion is 0.64 Å.^18^ Clearly, R_I_ depends on the ionic radii difference between the *B*–Ta cations. It increases considerably in the Sc-phase.

**Table 5:** Peak Intensity Ratio of the Mn^4+^ R-Line (ZPL) to the Most Intense Vibronic Sideband (ν_6_) in Ba*B*TaO_6_ (*B* = Y, Lu, Sc) at *T* = 12 K^a^

Perovskite	Peak intensity ratio R_I_ = ZPL/ν_6_	*R*_*B*^3+^_–*R*_*Ta*^5+^_ (Å)	*τ*_*av*_ (ms)
Ba_2_YTaO_6_	0.11	0.26	1.05
Ba_2_LuTaO_6_	0.13	0.221	0.96
Ba_2_ScTaO_6_	0.40	0.105	0.87

a *R*_*B*^3+^_–*R*_*Ta*^5+^_ is the ionic radii difference between the *B*–Ta cations. *τ*_*av*_ is the emission lifetime at *T* = 12 K.

It should be noted that the octahedral site that is occupied by the Mn^4+^ ion in Ba*B*TaO_6_ (*B* = Y, Lu, Sc) is centrosymmetric not only with respect to the nearest neighbor oxygen ligand coordination but also with respect to the next nearest neighbor cations. In consequence, the R-line is expected to be a magnetic dipole transition with small oscillator strength. The vibronic sidebands which are induced by odd-parity vibrations are electric-dipole allowed and have a much larger oscillator strength. Therefore, R_I_ in these compounds is expected to be low. The low R_I_ value in the Y and Lu-phases is in accordance with the expectation. However, R_I_ is three-times higher in the Sc-phase due to the R-line gaining significant intensity. The interpretation of this result is provided in the following.

For the double perovskite compounds, Barnes et al. have quantified the degree of cation ordering with the long-range order parameter (*LRO*):^34^
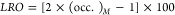
3where (occ.)_*M*_ is the fractional occupancy of the Ta cation on the octahedral site that is predominantly occupied. For Ba_2_ScTaO_6_, *LRO* = 51%.^35^ This means that the site occupied preferentially by Ta^5+^ contains 75% Ta^5+^ and 25% Sc^3+^. For isostructural Ba_2_YNbO_6_, *LRO* = 100%.^34^ Since the ionic radii of Nb^5+^ and Ta^5+^ are the same in six-coordination, the *LRO* of Ba_2_YTaO_6_ = 100%. This implies that the Ta^5+^ site is fully occupied by Ta^5+^ ion.

The cationic mixing model permits the rationalization of results of Table 5. In the double perovskite structure, the Sc–Ta mixing removes the inversion symmetry about the Mn^4+^ ion from the next nearest neighbor cations. The higher R_I_ value in the Sc-phase is consistent with the R-line gaining oscillator strength due to relaxation of the parity selection rule. The low and similar values of R_I_ in the Y- and Lu-phases is due to the lack of significant mixing between the Y/Lu–Ta cations. Nonetheless, the ratio is a bit higher in Ba_2_LuTaO_6_ where the ionic radii difference is smaller (Table 5). Further, the perturbation caused by the cationic mixing accounts for the relatively larger excitation and emission bandwidths in Ba_2_ScTaO_6_ (Figures 4 and 6). The cationic mixing is a favorable intrinsic defect in the double perovskite structure.

The lifetime of the Mn^4+2^E →^4^A_2_ emission transition, which was measured at *T* = 12 K, is assembled in Table 5. Even at low temperatures, the decay curves deviated from being single exponential. This suggests the presence of multiple Mn^4+^ sites presumably due to the charge compensation mechanism. An average lifetime was calculated using the relationship, . The variation of the lifetime with the *B*-cation is consistent with the expectation of the degree of mixing between the *B*–Ta cations. The faster radiative lifetime in Ba_2_ScTaO_6_ is due to the higher oscillator strength of the emission transition due to the considerable Sc–Ta cationic mixing which relaxes the parity section rule. The lifetime in the Lu-phase is faster than that of the Y-phase which is consistent with the smaller difference between the ionic radii of the Lu and Ta cations (Table 5).

In conclusion, the spectroscopic properties of the Mn^4+^ ion have been studied in a series of isostructural double perovskite compounds with the general formula, Ba_2_*B*TaO_6_ (*B* = Y, Lu, Sc). The energy and intensity of the Mn^4+2^E → ^4^A_2_ emission transition (R-line) are found to depend on the covalence of the [*B*TaO_6_] perovskite framework and on the degree of order between *B*–Ta cations. The R-line energy decreases as the covalence of the perovskite framework increases, consisting of corner shared *B*O_6_ and TaO_6_ octahedrons. This agrees with the expectation of the Tanabe-Sugano diagram for ions with d^3^ electronic configuration. The covalence of the perovskite framework is dependent on the electronegativity and the electronic configuration of the *B-*cation. The intensity of the R-line was found to be dependent on the degree of order between the *B*–Ta cations. The degree of cationic mixing depends on the ionic radii difference between these cations. Significant mixing occurs in the Sc-phase which lowers the local symmetry about the Mn^4+^ ion. In consequence, the R-line gains considerable intensity in Ba_2_ScTaO_6_ due to relaxing of the parity selection rule.

It should be noticed that if the coordination octahedron is perfect among the series of compounds (this is a rather rare case), then degree of covalence defining the R-line energy is correlated with the electronegativity of the coordinating cations without other structural interferences (such as decreased hybridization due to octahedral angles deviating from 90°). Based on an in-depth analysis of literature data, this information is the first communication of mentioned above phenomenon in the Mn^4+^-based red phosphors with double perovskite structures. From the fundamental point of view, our results provide evidence that the spectroscopic data of the Mn^4+^ ion (excitation, emission, and lifetime) can probe and can be an alternative and effective method for investigating the covalence as well as intrinsic disorder of the host lattice. The work also shows that the knowledge gained from the systematic study of the host–Mn^4+^ interaction can serve as a guide to practical improvement and tuning to desired values the key parameters of phosphors.

## Experimental Section

Since we are interested in correlating the optical properties to the degree of order between *B*–Ta cations, the samples were prepared under identical conditions. This is because the degree of order between *B*–Ta cations depends on the synthesis condition. Specifically, the temperature and time of synthesis are of importance.

We followed the procedure of Eng et al. to synthesize the double perovskite compounds.^23^ In the first step, the compound (Y, Lu, Sc)(Ta_0.99_Mn_0.01_)O_4_ was synthesized by a flux solid-state reaction technique. The starting materials, (Y, Lu, Sc)_2_O_3_, MnCO_3_, and Ta_2_O_5_ are mixed with Li_2_SO_4_ and heated to 1200 °C in air for a period of 10 h.^35^ The resulting powder was washed in hot water to remove the Li_2_SO_4_ flux. The Ba_2_*B*TaO_6_:Mn^4+^ (*B* = Y, Lu, Sc) compounds were synthesized via the following reaction: (Y, Lu, Sc)(Ta_0.99_Mn_0.01_)O_4_ + 2BaCO_3_→ Ba_2_(Y, Lu, Sc)(Ta_0.99_Mn_0.01_)O_6_ + 2CO_2_.

The precursor (Y, Lu, Sc)(Ta_0.99_Mn_0.01_)O_4_ was mixed with the appropriate amount of BaCO_3_ and the mixture heated in air to 1300 °C for a period of 10 h in alumina crucible. The resulting powder had a white body color.

The phase purity of the samples was determined by X-ray diffraction (XRD) performed in the range 2θ = 10°–70° with a step 2θ = 0.02° using a Rigaku Miniflex 6 G diffractometer. Cu Kα1 radiation of 1.541 Å wavelength was used. The excitation, emission, and lifetime data were obtained at low temperatures using a closed cycle He system of an Advanced Research Systems cryostat (CS204AE) using Edinburgh FS5 spectrofluorometer.

## Supporting Information Available

Transparent Peer Review report available (PDF)
