# Supplementary material for: Effect of Covalence and Degree of Cation Order on the Luminous Efficacy of Mn4+ Luminescence in the Double Perovskites, Ba2BTaO6 (B = Y, Lu, Sc)
Source: J Phys Chem Lett. 2024 Apr 10;15(15):4175–84. doi: 10.1021/acs.jpclett.4c00205 (PMC11033934; doi:10.1021/acs.jpclett.4c00205)
Supplement: Supplementary file 1 — jz4c00205_si_001.pdf [file jz4c00205_si_001.pdf]

jz-2024-00205m.R1

Name: Peer Review Information for "Effect of covalence and degree of cation order on the luminous efficacy of Mn⁴⁺ luminescence in the double perovskites

Ba₂<i>B</i>TaO₆ (<i>B</i>=Y, Lu, Sc)"

First Round of Reviewer Comments

Reviewer: 1

Comments to the Author

In this report, Mn4+ in the double perovskites, Ba2BTaO6 (B=Y, Lu, Sc), were synthesized. Moreover, the spectroscopic properties of them are investigated in detail. A comparison of the properties influence of covalent bonding and the degree of order between the B3+-Ta cations on the energy and intensity of the Mn4+ 2E→4A2 emission transition (R-line). However, I could not find more interesting information in this work as the similar results have been concluded in other papers from M. G. Brik.

Reviewer: 2

Comments to the Author

The authors present a study on the spectroscopic properties of Mn4+ ions within a set of isostructural double perovskites, namely Ba2BTaO6 (where B=Y, Lu, Sc). The discussion revolves around the correlation between the emission energies and intensities of Mn4+ R lines and two key factors: the covalence of the [BTaO6] perovskite framework and the degree of order among B-Ta cations. The findings underscore the utility of spectroscopic data on Mn4+ ions in solids as a robust tool for probing the fundamental aspects of materials, specifically gauging covalence and disorder. The paper demonstrates a commendable organization and writing style. Therefore, I recommend its publication in JPCL after minor revisions:

(1) It is advisable to compute Racah parameters B and C to bolster the authors' understanding of the covalence within the [BTaO6] perovskite framework.

(2) The authors are encouraged to explore whether there is any discernible trend in the energies of the nu3, nu4, and nu6 phonon modes concerning the B composition.

Author's Response to Peer Review Comments:

Dear Editor and Reviewers,

We appreciate all the comments we received from the Editor and Reviewers. They allowed to improve our submission. We carefully addressed each of the remarks in preparing the corrected version of our manuscript. Since no significant changes in the text was required, the minor corrections are not visible in the present version of the manuscript. We believe that you will find the corrected manuscript acceptable for publication in the The Journal of Physical Chemistry Letters.

Below we present in detail how we have addresses each of the Reviewers' remark. For your convenience, all significant changes (excluding corrected grammar and typos) are highlighted in yellow.

Reviewer #1

General comment:

Recommendation: This paper is not recommended because it does not provide new physical insights.

Comments:
In this report, Mn4+ in the double perovskites, Ba_2BTaO_6 (B=Y, Lu, Sc), were synthesized. Moreover, the spectroscopic properties of them are investigated in detail. A comparison of the properties influence of covalent bonding and the degree of order between the B3+-Ta cations on the energy and intensity of the Mn^{4+} 2E→4A2 emission transition (R-line). However, I could not find more interesting information in this work as the similar results have been concluded in other papers from M. G. Brik.

Authors Reply: We appreciate the reviewer's comment. We apologize that we did not explicitly indicate the novelty element in the earlier version of the technical manuscript. In particular the novelty is that if the octahedron is perfect among the series of compounds (this is rare) then covalence defining the R-line energy is correlated with parameters such as electronegativity of the coordinating cations without other structural interferences (such as decreased hybridization due to octahedral angle deviating from 90 degree). In the papers on double perovskite phosphors with Mn^{4+}, this is the first of its kind, although, as the continuation, it make use to some extent on the our previous works achievements.

This has been rectified by including appropriate information in both the abstract and conclusions:

1. In the Abstract:

The purpose of this work is to propose for first time an explanation of these effects in the considered double perovskites.

2. In Conclusion:

It should be noticed that if the coordination octahedron is perfect among the series of compounds (this is a rather rare case) then degree of covalence defining the R-line energy is correlated with the electronegativity of the coordinating cations without other structural interferences (such as decreased hybridization due to octahedral angles deviating from 90 degrees). Based on an in-depth analysis of literature data this information is the first communication of mentioned above phenomenon in the Mn^{4+}-based red phosphors with double perovskite structures.

Reviewer #2

Comment 1:

Recommendation: This paper is publishable subject to minor revisions noted. Further review is not needed.
Authors Reply: We appreciate the reviewer's positive recommendation.

Comment 2:

The authors present a study on the spectroscopic properties of Mn4+ ions within a set of isostructural double perovskites, namely Ba2BTaO6 (where B=Y, Lu, Sc). The discussion revolves around the correlation between the emission energies and intensities of Mn4+ R lines and two key factors: the covalence of the [BTaO6] perovskite framework and the degree of order among B-Ta cations. The findings underscore the utility of spectroscopic data on Mn4+ ions in solids as a robust tool for probing the fundamental aspects of materials, specifically gauging covalence and disorder. The paper demonstrates a commendable organization and writing style. Therefore, I recommend its publication in JPCL after minor revisions:

Authors Reply: We appreciate the reviewer's positive evaluation.

(1) It is advisable to compute Racah parameters B and C to bolster the authors' understanding of the covalence within the $[BTaO_6]$ perovskite framework.

Authors Reply: Thanks a lot for interesting suggestion. We performed and added to the manuscript the estimations of the B and C values, along with the beta_1 parameter. Those estimated values are in line with the earlier included to manuscript discussion.

The above comment has been taken into account by including the following information in section 3.3:

In Table 4 are assembled the values of the Racah parameters B, C (along with the crystal field strength Dq) for the Mn^{4+} ion. The parameters were estimated from the excitation and emission spectra of the considered double perovskites with the help of standard equations for the energy levels of the d^3 electron configuration [33]. In addition, the values of the new nephelauxetic parameter $\beta_1 = \sqrt{\left(\frac{B}{B_0}\right)^2 + \left(\frac{C}{C_0}\right)^2}$ [9] were calculated; B_0 = 1160 cm^{-1}, C_0= 4303 cm^{-1} are the Racah parameters for the free Mn^{4+} ion [34].

Table 4: Spectroscopic parameters for the Mn^{4+} ions (all in cm^{-1}) in $BaBTaO_6$ (B=Y, Lu, Sc) double perovskites.

	B	*C*	*Dq*	β_1	*Reference*
Ba_2YTaO_6	*1019*	*2560*	*1923*	*1.061*	*This work*
Ba_2LuTaO_6	*971*	*2627*	*1899*	*1.036*	*This work*
Ba_2ScTaO_6	*756*	*2958*	*1785*	*0.947*	*This work*

The β_1 parameter reflects the reduction of the Racah parameters caused by covalent bonding when the ion is embedded in a host compound [9]. The smaller is the value of β_1, the higher is the covalence of the Mn-O bonding. The data indicates that the Mn-O bonding covalency increases in the sequence, Y<Lu<Sc. This is exactly as derived from the structural peculiarities of these double perovskite frameworks and influence of the second cation on the Mn^{4+} spectroscopic properties.

(2) The authors are encouraged to explore whether there is any discernible trend in the energies of the nu3, nu4, and nu6 phonon modes concerning the B composition.

Authors Reply: Regarding reviewer comment on the possible trend across the lattice modes: that is does not seem to be a trivial question, the energies (or frequencies) of the normal modes depend on the interatomic distances and chemical bonds stiffness and the precise analysis of these effects is beyond the manuscript scope.

jz-2024-00205m.R2

Name: Peer Review Information for "Effect of Covalence and Degree of Cation Order on the Luminous Efficacy of Mn⁴⁺ Luminescence in the Double Perovskites Ba₂<i>B</i>TaO₆ (<i>B</i>=Y, Lu, Sc)"

Second Round of Reviewer Comments

Reviewer: 1

Comments to the Author

The revised version could be accepted for publication.

Author's Response to Peer Review Comments:

In current version we take account all required technical requirements (changes of structure, additional affiliations information, new TOC etc.)

Editor: The Journal of Physical Chemistry

Dear Editor,

I'm sending our second time revised manuscript: **Effect of Covalence and Degree of Cation Order on the Luminescence of Mn^{4+} in the Double Perovskites, Ba_2*B*TaO_6 (*B*=Y, Lu, Sc) on the Phosphor Luminous Efficacy**, with a request to consider the possibility of publishing in *The Journal of Physical Chemistry*. In current version we take account all required technical requirements (changes of structure, additional affiliations information, new TOC etc.)

After performed improvements we believe that second time revised manuscript is suitable for publication in The Journal of Physical Chemistry (see my comment above). Thank you.

Sincerely yours, Michał Piasecki

Prof. M. Piasecki (on behalf of all coauthors)
